# Advancing Cervical Cancer Prevention Equity: Innovations in Self-Sampling and Digital Health Technologies Across Healthcare Settings

**DOI:** 10.3390/diagnostics15091176

**Published:** 2025-05-06

**Authors:** Michelle Gomes, Elena Provaggi, Andrea Barnabas Pembe, Adeola Olaitan, Aleksandra Gentry-Maharaj

**Affiliations:** 1 Department of Global Health and Development, The London School of Hygiene and Tropical Medicine (LSHTM), London WC1E 7HT, UK; michelle.gomes@lshtm.ac.uk; 2 Anne’s Day Ltd. (Daye), London SE16 4DG, UK; elena.provaggi@yourdaye.com; 3 Department of Obstetrics and Gynecology, Muhimbili University of Health and Allied Sciences (MUHAS), Dar es Salaam 11103, Tanzania; andreapembe@yahoo.co.uk; 4 Department of Women’s Cancer, EGA Institute for Women’s Health, University College London, London WC1E 6DD, UK; adeola.olaitan@nhs.net; 5 MRC Clinical Trials Unit at UCL, Institute of Clinical Trials and Methodology, University College London, London WC1V 6LJ, UK

**Keywords:** cervical cancer (CC), human papillomavirus (HPV), screening, self-sampling, low- and middle-income countries (LMICs), FemTech

## Abstract

Cervical cancer causes 350,000 deaths annually, with 90% occurring in low- and middle-income countries (LMICs), despite being largely preventable through vaccination and screening. This review examines innovative approaches to address screening coverage gaps worldwide, analysing both established programmes in high-income countries and implementation strategies for LMICs. Self-sampling technologies demonstrate significant potential to improve the uptake of cervical screening, thereby improving cervical cancer prevention compared to traditional methods, particularly benefiting underserved populations across all healthcare settings. Among self-collection devices, vaginal brushes achieve sensitivity of 94.6% (95% CI: 92.4–96.8) for HPV detection, while novel approaches like the tampon show promising results (sensitivity 82.9–100%, specificity 91.6–96.8%) with high user acceptability. Implementation strategies vary by healthcare context, with high-income countries achieving success through integrated screening programmes and digital solutions, while LMICs demonstrate effective adaptation through community-based distribution (20–35% uptake) and innovative delivery methods. In resource-limited settings, self-sampling increases participation through enhanced patient comfort and cultural acceptability, while reducing costs by 32–48%. Progress toward WHO’s cervical cancer elimination goals require careful consideration of local healthcare infrastructure, cultural contexts and sustainable financing mechanisms. Future research priorities include optimising self-sampling technologies for sustainability and scalability, developing context-specific implementation strategies and validating artificial intelligence applications to enhance screening efficiency across diverse healthcare settings.

## 1. Introduction

FemTech represents a rapidly evolving sector of technology-driven solutions transforming cervical cancer prevention through artificial intelligence (AI)-enabled diagnostics, smart self-sampling devices and integrated digital platforms [1,2]. This review provides a comprehensive overview of global cervical cancer screening strategies and provides evidence on self-sampling technologies and digital innovations (e.g., AI integration, point-of-care platforms) as novel solutions to address disparities in screening accessibility across healthcare systems, a critical gap in the existing literature. It further delineates the implementation strategies for self-sampling in high-income countries (HICs) compared with low- and middle-income countries (LMICs) to provide insights into how cervical cancer screening equity can be achieved.

Human papillomavirus (HPV) infection is extremely common, with an estimated 80% of sexually active people acquiring at least one HPV type during their lifetime [3,4]. While most infections clear naturally within two years, persistent infection with high-risk HPV types causes 99.7% of cervical cancer cases, making HPV detection an important screening strategy [5,6]. In 2022, there were 660,000 new diagnoses and 350,000 cervical cancer deaths globally [7,8].

This preventable disease disproportionately affects LMICs, which account for 90% of cervical cancer mortality [9,10,11]. The burden varies significantly by region, with age-standardised incidence rates ranging from 75 cases per 100,000 women in some African countries to less than 10 per 100,000 in many HICs [11]. People with compromised immune systems, particularly those living with HIV, face a significantly higher risk of persistent HPV infection and progression to cervical cancer. Women living with HIV are six times more likely to develop cervical cancer compared to the general population [12]. This elevated risk is compounded in LMICs by the dual challenge of a high prevalence of HIV and inequitable access to essential preventive measures, including HPV vaccination and cervical cancer screening programmes [13,14].

The experience from the United Kingdom illustrates both progress and persistent challenges in cervical cancer control. While comprehensive national screening programmes contributed to a 25% decrease in incidence rates since the early 1990s, data from the past decade show a 4% increase suggesting evolving risk factors and potential gaps in screening coverage [15,16]. This trend has been exacerbated by the COVID-19 disruptions, with screening participation declining significantly. Some areas of London now report screening uptake rates as low as 48%, reflecting widespread disengagement with screening services [17,18].

**Figure 1:**
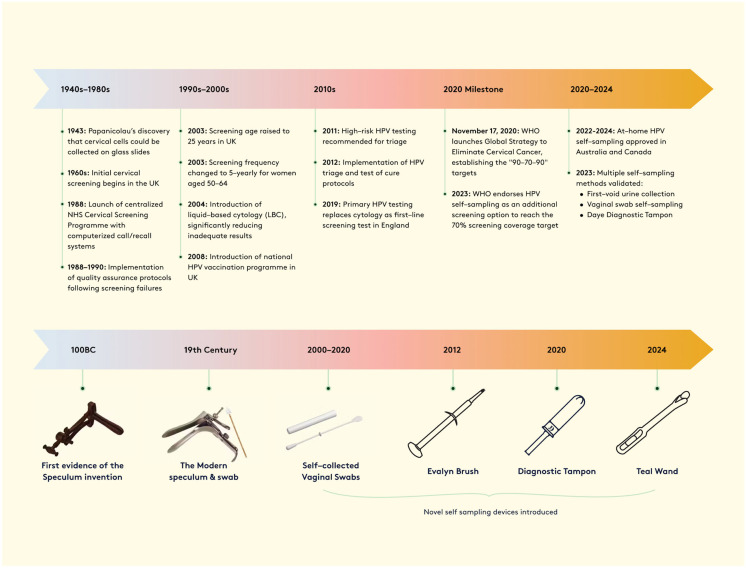
Timeline of cervical screening, 1940s–2024. The upper panel shows key policy and technological developments in cervical screening programmes, highlighting major milestones in the UK and globally. The lower panel illustrates the progression of screening devices from ancient speculums to modern self-sampling methods, demonstrating the advancement toward patient-centred screening approaches.

Screening detects asymptomatic precancerous lesions, which if undetected and untreated can put women at risk of developing cervical cancer. In 2023, the National Health Service announced a plan for cervical cancer elimination by 2040 (defined as fewer than 4 cases per 100,000 women), which aligns with the World Health Organization (WHO) global 90–70–90 initiative, which aims for 90% HPV vaccination coverage, 70% screening coverage and 90% treatment of precancerous lesions by 2030 [9,19]. Achieving this goal requires enhanced HPV vaccination coverage, increased screening participation and innovative solutions adaptable to diverse healthcare settings [20,21,22,23,24]. Scotland demonstrates the potential success of comprehensive prevention strategies, achieving near elimination through high vaccination uptake and effective screening programmes [25]. The prevention of cervical cancer stands at a critical transition point. While HPV vaccination programmes are widespread in HICs, their full impact on mortality rates will take decades to realise due to the extended latency between infection and cancer development [26,27]. Meanwhile, the evolution from traditional cytology to HPV-based screening (see Figure 1) offers enhanced detection capabilities, though implementation challenges persist, especially in resource-limited settings [28,29].

### 1.1. Prevention Strategies

#### 1.1.1. Primary Prevention

HPV vaccination demonstrates exceptional efficacy, with the Swedish nationwide study (1.7 million women) showing a 90% reduction in cervical cancer incidence among girls vaccinated before age 17 over 2006–2017 [30]. Norwegian registry data showed a 82% reduction in CIN2+ in girls vaccinated at 12 years old [31]. Effectiveness significantly decreases when vaccination is given after age 17 [32]. A systematic review from 2023 demonstrated vaccine effectiveness ranging from 74 to 93% for ages 9–14 versus 12–90% for ages 15–18, highlighting the critical importance of vaccination at an early age [33]. The UK has adopted a single-dose HPV vaccine approach in everyone under the age of 25 except in the immunocompromised. If all countries adopted this approach, then it may improve accessibility and equity as well as affordability. The implementation of WHO’s 90% vaccination coverage strategy poses significant challenges in LMICs due to infrastructure limitations and cost barriers [34,35]. Historical controversies surrounding vaccine trials and implementation programmes have created enduring trust issues in some regions, particularly in India and Sub-Saharan Africa [36]. These challenges underscore the importance of ethical implementation practices, community engagement and cultural sensitivity in vaccination programmes.

#### 1.1.2. Secondary Prevention

The evolution of screening methods marks a significant advancement in cervical cancer prevention, transitioning from traditional cytology to HPV-based approaches [37,38]. While HICs maintain screening coverage above 60%, LMICs struggle with rates as low as 20%, highlighting the need for resource-appropriate solutions [39]. Secondary prevention through HPV testing has emerged as superior to cytology for cervical screening [40,41].

#### 1.1.3. Emerging Technologies

WHO’s 2021 guidelines recommended HPV DNA testing as the preferred screening method for women aged 30–49 years, with 5–10-year intervals [42]. Implementation success varies globally, requiring careful consideration of local healthcare infrastructure, resource availability, population-specific needs and healthcare system capacity [6,43].

Self-sampling represents a transformative approach to cervical cancer screening, particularly for underserved populations at elevated risk of HPV infection [44,45]. This review examines how innovative technologies and implementation strategies can bridge disparities in screening access. We analyse the potential of self-sampling and FemTech solutions to overcome traditional barriers, with particular focus on their application in resource-limited settings where conventional screening methods remain challenging to implement.

## 2. Understanding HPV and Cervical Carcinogenesis: A Foundation for Prevention

Understanding the relationship between HPV infection and cervical cancer development is crucial for developing effective prevention strategies and implementing appropriate screening programmes [6,46,47]. Persistent infection with high-risk HPV is the primary causative agent of cervical cancer, with high-risk types 16 and 18 being responsible for approximately 70% of cases globally, while types 31, 33, 45, 52 and 58 contribute another 20% [48,49]. The global prevalence of HPV16 and HPV18 in women with normal cytology is 3.2% and 1.4%, respectively [50,51].

HPV causes cervical cancer through the persistent infection of epithelial cells, where the continued expression of the viral oncoproteins E6 and E7 disrupt cell cycle regulation, leading to genomic instability and malignant transformation [52,53]. This process can result in precancerous lesions, graded as Cervical Intraepithelial Neoplasia (CIN1–3), with CIN2/3 requiring treatment to prevent progression to cancer [54,55]. Early detection through screening and colposcopy examination is crucial for identifying and managing CIN [56].

### 2.1. HPV Vaccination

Prophylactic HPV vaccines have demonstrated efficacy, achieving up to 90% reduction in HPV infections and 85% reduction in high-grade cervical lesions among vaccinated cohorts aged 13–24 years [57]. Evidence suggests extending screening intervals for vaccinated populations, with recommendations to start screening vaccinated women at age 30 instead of the currently recommended age of 25 [58]. However, even the broadest 9-valent vaccine does not protect against all oncogenic HPV types, necessitating continued screening programmes [59,60]. While vaccination coverage is increasing globally, uptake remains suboptimal in many regions, and women aged 35 and older, who were not eligible for vaccination programmes, remain at risk and require regular screening [61,62].

Recent evidence supports WHO’s simplified HPV vaccination schedules: one dose for ages 9–14 years, one or two doses for ages 15–20 years and two doses for those over 21 years. This optimisation reduces costs by 30% while maintaining efficacy in younger age groups [63,64].

Global vaccination rates declined significantly, with studies showing coverage reductions of 42% in Italy and decreased uptake from 89.92% to 69.59% during the pandemic in US border communities [65]. These disruptions are projected to increase cases of cervical cancer and precancerous lesions over the coming decades, necessitating urgent recovery strategies [66].

### 2.2. Evolution of Cervical Cancer Screening Methods

The transition from traditional cytology to HPV-based screening represents a significant advancement in cervical cancer detection. HPV testing demonstrates superior detection of precancerous lesions with 98.1% sensitivity (95% CI: 96.3–96.7) for CIN3+, compared to cytology’s 48.5% sensitivity (95% CI: 44.0–53.0) [67]. While HPV testing shows slightly lower specificity (94.4%, 95% CI: 94.1–94.7) than cytology (97.9%, 95% CI: 97.8–98.1), new molecular technologies offer improved specificity without compromising sensitivity [67].

Emerging molecular approaches such as mRNA-based tests specifically detect active HPV infections by identifying viral oncogene expression, reducing false positives from transient infections [68]. Next-generation sequencing platforms enable the simultaneous detection of multiple biomarkers, allowing for better risk stratification of HPV-positive women. These innovations help identify women most at risk of disease progression while reducing unnecessary referrals [69].

### 2.3. Innovative Vaccination and Screening Approaches in High-Income Countries

High-income countries have pioneered different approaches to implementing HPV-based screening and self-sampling programmes, providing valuable insights for global cervical cancer prevention strategies. Australia and the UK have demonstrated the successful integration of vaccination and screening programmes. Australia’s comprehensive approach, achieving 80% vaccination coverage and implementing primary HPV screening, projects cervical cancer elimination by 2028, with a 92% reduction in high-grade abnormalities among women vaccinated before age 15 [26,70]. The UK’s strategy complements its screening programme, contributing to a 25% decrease in cervical cancer incidence since the 1990s [71].

European countries have led self-sampling innovations. The opt-in model in the Netherlands achieved 16% self-sampling uptake by 2020, while Sweden’s direct-mailing approach during COVID-19 increased coverage from 54% to 60% in one year, reaching Europe’s highest uptake with screening of 83% [72,73]. Both countries maintain sustained screening coverage above 70% through automated testing platforms and standardised protocols, particularly benefiting under-screened populations [72,73].

### 2.4. Prevention Strategies in Middle- and Low-Income Countries

Middle-income countries have developed effective hybrid approaches to cervical cancer prevention, exemplified by Malaysia and Thailand’s integration of national vaccination programmes with phased HPV-based screening implementation [74]. These programmes achieve 85–90% vaccination coverage through school-based delivery systems while expanding screening access via public–private partnerships [75].

WHO’s pragmatic approach for LMICs focuses on high-precision HPV testing with two lifetime screens at ages 35 and 45, balancing effective coverage against resource constraints [76]. Rwanda’s successful implementation demonstrates the potential of this approach, achieving 93% HPV vaccination coverage among girls aged 11–12 and screening 329,000 women between 2013 and 2016 [77].

Successful programmes utilise community health worker networks, mobile clinics and partnerships with local religious leaders while integrating self-sampling options to optimise resource utilisation [78]. This comprehensive approach enables LMICs to maximise precancerous lesion detection during peak risk periods while maintaining cost-effectiveness within resource-limited healthcare systems [74].

## 3. Implementation Barriers and Economic Impact of Cervical Screening in LMICs

The implementation of cervical cancer prevention programmes in LMICs faces significant systemic challenges that impact both healthcare delivery and economic outcomes. A WHO analysis (2022) of 45 African countries revealed that only 15% have universal health coverage schemes for cervical cancer screening, resulting in significant out-of-pocket expenditure for families [79]. Screening rates in LMICs remain critically low at 5%, exemplified by Nigeria where only 8.7% of 60.9 million at-risk women undergo screening, with even lower rates in rural areas [80].

The economic implications are substantial yet promising: every dollar invested in cervical cancer prevention yields a 3.20 USD return through improved health outcomes and productivity gains [81]. HPV-based screening programmes demonstrate high cost-effectiveness, with an incremental cost-effectiveness ratio of 569 USD per quality-adjusted life year gained [82].

### Innovative Implementation Strategies in LMICs

Several countries have developed effective solutions to increase screening coverage through innovative integration with existing healthcare services and novel delivery approaches. Rwanda has successfully integrated cervical screening with HIV/AIDS care services, with studies showing increased screening rates among HIV-positive women through these integrated services [77]. Ethiopia’s screening coverage remains very low at less than 2% nationally, while Zambia has achieved about 26% population-level coverage through HIV programme integration [83,84]. Mobile screening units and task-shifting strategies have been implemented in Rwanda to reach women in remote areas, with the country screening nearly 95,000 women and achieving 91% treatment rates for those testing positive [85]. This represents coverage of about 2.2% of the total at-risk population. This highlights the significant gap remaining to achieve WHO’s target of 70% screening coverage.

International support has been crucial, with Gavi, The Vaccine Alliance committing 600 million USD for HPV vaccination and the Global Fund reaching over 1 million women through integrated HIV screening programmes [86]. Many LMICs currently rely on visual inspection methods—direct inspection of the cervix to identify macroscopic morphological abnormalities—with typically low specificity (visual inspection with acetic acid (VIA)/visual inspection with Lugol’s Iodine (VILI)) due to resource constraints. These approaches, however, offer advantages of immediate results and same-day treatment [87]. The emergence of HPV self-sampling technologies presents an opportunity to implement more sensitive molecular testing while maintaining community-based screening benefits, which in some settings will be followed up by VIA/VILI and in some contexts referral to colposcopy (depending on the infrastructure available, based on country).

## 4. Enhancing Screening Participation Through Self-Sampling: Evidence and Implementation

Self-sampling represents a transformative approach to cervical cancer screening, addressing traditional barriers while improving accessibility for underserved populations through patient-centred collection methods. Cervical screening coverage in England has declined, with only 69.9–71.2% of eligible women screened within the recommended interval [88,89]. Barriers to screening include discomfort, embarrassment, time constraints and procedure-related anxiety [90]. Lower uptake is associated with younger age, ethnic minority background and socioeconomic deprivation [91]. Women who have experienced sexual abuse are less likely to attend screening [92]. Studies show that transgender men and non-binary people have significantly lower cervical screening uptake, with only 58% of those eligible having been screened [93]. Research indicates that transgender men are 37% less likely to be current with cervical screening compared to cisgender patients and ten times more likely to have inadequate test results [94].

The YouScreen trial in London (*n* = 8338) validated self-sampling’s effectiveness in increasing participation, achieving 56% uptake through GP practices compared to 13% via direct mailing. The approach showed particular success among ethnic minorities (64% coverage) and socioeconomically deprived populations (60% coverage). The trial design included offering self-sampling kits to over 27,000 under-screened women aged 25–64 across 133 GP practices in North and East London, with 8338 participants returning samples. Notably, half of those who returned self-samples were at least two years overdue for screening, underscoring the trial’s effectiveness in reaching historically underserved populations [95].

Implementation strategies for self-sampling vary in effectiveness, with community-based distribution through pharmacies and health centres achieving higher uptake (20–35%) compared to direct mailing methods (8–25%) [95]. The HPValidate study was designed to address gaps in cervical screening participation, particularly among under-screened populations who have never or rarely attended screening. By evaluating three self-sampling devices (Evalyn Brush, Rovers Medical, Lekstraat, The Netherlands; FLOQswabs COPAN Group, Murrieta, CA, USA; and Aptima Multitest, Hologic, Marlborough, MA, USA) and two HPV testing platforms (Cobas and Aptima), the study provided critical insights into the accuracy, practicality and acceptability of self-sampling for integration into the NHS Cervical Screening Programme. The UK HPValidate study validated multiple device-test combinations (three different collection devices: Evalyn Brush (Rovers Medical), Self-Vaginal FLOQswabs (COPAN Group) and Aptima Multitest (Hologic), demonstrating strong user preference (85%) for having self-sampling as an option alongside traditional screening [96].

Economic analyses demonstrate the cost-effectiveness of self-sampling in low-resource settings. A 2023 study in Sikkim, India found that HPV self-testing cost 15.3 USD per woman screened compared to 19.2 USD for traditional screening, representing a significant cost reduction [97].

Successful implementation in LMICs requires tailored delivery approaches combining clinic-based and home-based methods. Community health workers facilitate education and sample collection through door-to-door visits, while trusted community leaders address health literacy through visual instructions [97]. Self-sampling swabs can maintain sample integrity at room temperature for up to two weeks, eliminating the need for cold-chain logistics and simplifying transportation in low-resource settings [98]. Digital platforms enable result communication where infrastructure permits, as demonstrated by the PRESCRIP-TEC project across Bangladesh, India, Uganda and Slovakia. Digital platforms enable result communication where infrastructure permits, as demonstrated by the PRESCRIP-TEC project across Bangladesh, India, Uganda and Slovakia, which implemented a multi-faceted strategy combining community engagement, mobile health interventions and artificial intelligence decision support systems to increase cervical cancer screening uptake [99].

Self-sampling is endorsed by WHO because of its potential to increase screening coverage among underserved populations, though success depends on standardised procedures, comprehensive follow-up protocols and context-specific implementation strategies that consider local healthcare infrastructure and economic conditions [45].

## 5. Evolution and Performance of Self-Sampling Technologies in Cervical Screening

Cervical self-sampling methods have advanced in accuracy and improved in accessibility, with various collection methods demonstrating increased accuracy and accessibility while addressing traditional barriers to participation [100]. There are multiple devices with diverse design features as illustrated in Table 1.

Performance metrics from meta-analyses show that self-collected samples have slightly lower detection rates compared to clinician collection, with sensitivity reduced by 14% (95% CI: 9–20%) and specificity by 11% (95% CI: 8–15%) [101]. However, newer PCR-based assays demonstrate comparable accuracy, particularly with lavage devices and brushes [101].

The integration of AI enhances screening precision through advanced image analysis capabilities. Recent studies demonstrate AI algorithms achieving sensitivity and specificity ranging from 22 to 93% and 67 to 95%, respectively, in classifying visual inspection images [102,103]. Wu et al. (2024) highlight how AI-assisted digital microscopy platforms like CytoBrain can analyse digitised cervical samples with up to 78% efficiency in cell classification, reducing reliance on specialised personnel while maintaining diagnostic accuracy [104]. While these technologies show particular promise for resource-limited settings, large-scale validation in real-world conditions remains crucial for establishing clinical feasibility.

Recent advancements in point-of-care (POC) HPV testing offer promising solutions for cervical cancer screening in resource-limited settings. These tests aim to provide rapid results with high sensitivity and specificity, addressing barriers in traditional cytology-based screening [105,106]. The careHPV test demonstrates good performance, with sensitivity and specificity of 88% and 84% for CIN2+ detection [107]. New technologies like isothermal amplification and lateral flow detection enable low-cost, sample-to-answer HPV testing suitable for decentralised screening [20]. HPV-based screen-and-treat approaches have shown effectiveness in reducing cervical disease and over-treatment compared to visual inspection methods [108]. A study in Papua New Guinea found high acceptability and safety of an integrated POC HPV self-sampling and same-day treatment strategy. POC innovations are transforming screening accessibility in resource-limited settings [109]. Similar to innovations like the Hemex Health Gazelle platform for sickle cell disease, emerging point-of-care technologies for HPV testing demonstrate the potential for rapid, affordable screening with high accuracy, achieving results within minutes while maintaining laboratory-grade standards, thus addressing critical access barriers in resource-limited settings [110].

WHO has recently launched updated target product profiles for POC tests, emphasising the need for affordable, user-friendly devices suitable for low-resource settings [111]. These profiles aim to guide manufacturers in developing tests that meet specific performance and operational characteristics crucial for effective cervical cancer screening in diverse healthcare contexts [111].

**Table 1:** Comprehensive comparison of current self-sampling devices, highlighting key features [112,113,114,115,116,117,118,119,120,121,122,123,124,125,126,127,128,129,130,131,132,133,134,135,136,137,138,139,140].

Sampling Approaches and Devices				Other Features for Evaluation			
Device Type	Device Name (Company Name)	Key Features and Materials	Sensitivity and Specificity for HPV DNA	Sensitivity and Specificity for CIN2+	Concordance Between Self and Conventional Samples	Cost, Ease of Use and Comfort	Innovation and Performance Comparison
**Vaginal Swabs**	**FLOQSwab^®^** Company: COPAN Group Headquarters: Brescia, Lombardy, Italy US Operations: Murrieta, CA, USA Note: COPAN is a global company with manufacturing in Italy and the USA. Introduced in 2003.	Nylon strands are flocked onto the swab tip using electrostatic force, which allows much greater absorption and release of cells by capillary action.	Sensitivity: 93.8% dry samples, 96.3% wet samples. Specificity: 87.5% dry samples, 97.5% wet samples [112].	Sensitivity: 89% for CIN2+ [113], 91–93% for CIN3+ [118].	94–99% for HPV16/18 [116].	Easy to use [114,115,117]; not uncomfortable to use [118].	Innovative flocked surface for efficient cervicovaginal cell collection. VERA study [118] demonstrated its effectiveness for HPV testing and cervical cancer screening.
	**Qvintip (Aprovix)** Company: Aprovix AB City: Solna Country: Sweden Introduced in the 2000s. Commercialised with distribution in Europe, China and Russia.	Swab device with a 5 cm, 7 mm thick plastic head with grooves for cervicovaginal cell collection. Inserted, removed, and head transferred into a separate tube.	Sensitivity; 83.1% [119]; Specificity 51.3% [119]		81.8% (HPV test); 77.1% (Cytology) [119]	Easy to use [116,118,120,121]; comfortable to use [116]	Lower sensitivity than dry flocked swabs and wet Dacron swabs [112].
	**HerSwab (Eve Medical)** Company: Eve Medical Inc. City: Toronto, ON, Country: Canada Introduced 2010–2020, commercialised in Canada.	Curved tip with a soft, flexible design for gentle collection and an ergonomic handle for improved control.	Sensitivity: 75.0% [119]; specificity: 47.7% [119].	Sensitivity: 87.6% [122]; specificity: 58.1% [122].	74.8% (HPV test); 74.5% (cytology) [119].	3–8 Canadian dollars per device (if used within screening programmes); easy to use [112,123]; comfortable to use [123]. *(Studies were in relation to STI self- sampling, however still relevant to HPV self sampling.)*	Lower sensitivity than dry flocked swabs and wet Dacron swabs [112]. Dry samples should be processed as soon as possible (within 5–7 days), which may be a consideration if used in remote areas [124].
**Brush**	**Evalyn^®^ Brush (Rovers Medical Devices)** Company: Rovers Medical Devices B.V. City: Oss, North Brabant (NB) Country: The Netherlands Introduced in 2012. Commercialised in the UK, USA, Europe, Asia and Pacific.	Featuring flexible LDPE bristles, an ergonomic inserter with stopper wings for correct depth and a plunger mechanism for bristle extension and retraction. The user performs five rotations, each indicated by audible “clicks,” after which the sample is retracted into a protective casing.		96–99% for CIN3+ [125].	96.8% hrHPV; concordance between self-samples and physician-taken samples [126,127].	More expensive than Vina Brush; very easy; highly accepted; one study found Evalyn^®^Brush to be slightly more comfortable than FLOQSwabs™ [118].	Retractable device for sample preservation. Large clinical study in Dutch population (VERA Study); promising device for remote areas, due to analytical stability at room temperature and humidity for extended periods [118].
	**US2018078242A1** This is a US patent application number, not a company Invention filed in 2018. Not commercialised.	- A sampling tool connected to a plunger syringe via a flexible tube - Automatic rotation mechanism					Advantages: automatic rotation mechanism. The sampling brush is not exposed to vulva during insertion. Disadvantages: bulky as it is made from two components, which limits its portability compared to the other devices [117].
**Lavage**	**Delphi Screener (Rovers Medical Devices)** Company: Rovers Medical Devices B.V. City: Oss, North Brabant (NB) Country: The Netherlands Introduced in 2006. Commercialised in the UK, USA, Europe, Asia and Pacific.	The device is pre-filled with 3–5 mL saline solution. After insertion, the user will press the button plunger, compressing the spring while injecting the saline solution into the cervix. After three seconds, the user will release the button plunger, causing the spring to recoil while aspirating the saline solution back into the device.					Most efficient in collecting shed cells as it does not scrape the cervix directly, hence its utility is more towards HPV DNA molecular testing rather than cytology [117,128].
**Sponge**	**Kato device** Company: Taisei Kako Co., Ltd. City: Ibaraki, Osaka Country: Japan Introduced 2012–2015. Commercialised in Japan.	Soft sponge for gentle cervical sampling and a plastic handle for easy manipulation.			100% agreement with gynaecologist sampling but only 32.3% agreement for presence of endocervical cells [117,129,130].		For Pap specimen adequacy, Kato device sampling showed 100% agreement with gynaecologist sampling. Less effective for collecting EC/TZ (endocervical cells/transformation zone) cells than gynaecologist sampling: 68% of samples from Kato device were absent of EC/TZ cells, but present in gynaecologist sampling [117,129,130].
	**The Teal Wand™** Company: Teal Health, Inc. City: San Francisco, CA Country: USA Introduced in 2024. Not yet commercialised. Received FDA Breakthrough Device designation in 2024.	Retractable device made with soft material for cell collection. It features a marker to indicate proper insertion depth.	Clinical trial (SELF-CERV) ongoing [131,132].			Very easy to use [131,132].	Innovative retractable design for sample preservation.
**Non-woven**	**V-Veil UP2™** Company: V-Veil-Up Production SRL City: Calinesti, Arges Country: Romania Introduced in 2019. Commercialised in Europe.	Includes a 75 mm pocket made of non-woven hydrophilic polyethylene to harvest cells, proteins and DNA/RNA from the cervix, and a 120 mm applicator made of low-density polyethylene (LD-PE).	Sensitivity: 95.9% [132]. Specificity: 88.2% [132]		Good agreement.	Low cost; very easy to use; highly acceptable.	Innovative pocket design pocket design, which effectively retains genital secretions. Studies show it offers 1.67- and 1.57-fold detection rates of cervical HPV DNA and high-risk (HR)-HPV DNA by self-sampling with veil compared to clinician-collected cervical secretions by swab. High acceptability (≥96%), feasibility and satisfaction. Pitched as cost-effective alternative for LMICs [132].
**Cannula/** **Thin tube**	**Mia by XytoTest** Company: MEL-MONT Medical, Inc. City: Doral, FL Country: USA Introduced 2015–2017. Commercialised (Colombia, Mexico, Europe), CE marked the IVD directive 98/97/EC since 2017.	Features a highly adhesive, hypoallergenic USP medical-grade IV elastomer coating on its cell collection area for immediate cell collection upon insertion. Diameter of less than 8 mm and length of 14 cm.	Sensitivity: 95.7% [133,134]. Specificity: 91.7% [133,134].		k 0.86 (HPV); k 0.41 (Pap smear) [133,134].	Easy to use; highly acceptable [133,134].	Enables both HPV DNA and mRNA E6/E7 testing for triaging positive samples prior to referral for colposcopy (proteins associated with the progression of HPV infection to cervical cancer). Provides risk stratification. Suitable for LMICs.
	**Iune HPV Test Cannula** Manufacturer: QUIROSA, S.A. City: Artés, Cataluña Country: Spain Commercialised in Europe.	A “cannula” or thin tube-like device made of soft material.	Sensitivity: 90.9% [140]. Specificity: 84.6% [140]		k 0.73 (HPV) [140].		Moderate agreement with clinician-collected samples for HPV detection. Lower performance for cytology compared to FLOQSwab and Evalyn Brush [140].
**Tampon**	WO2002041785A1 This is a World Intellectual Property Organization (WIPO) patent publication number, not a company.	Cardboard applicator, organic/inorganic tampon. Handle adapted to allow it to serve as a screw-cap lid, once the device is inserted into a conical tube containing fixative or preservative. Overall length of the device is 15 cm. Length of the sheath is 13 cm when fully extended. Maximum width 1.5 cm.					
	**Daye Diagnostic Tampon** Company: Daye (Tampon Innovations Ltd.) City: London (Southwark) Country: UK Introduced in 2024. Commercialised in the US and UK.	The kit includes a tampon made of organic cotton and a Bio LDPE applicator, with the tampon measuring 4.75 cm in length and the applicator extending to 12.5 cm. Users are instructed to leave the tampon in place for at least 20 min to ensure optimal sample collection.	Sensitivity: 82.9 (72.4–89.9) [135]. Specificity: 91.6 (86.4–94.9) [135].			69 GBP; very easy; highly accepted [135].	Non-invasive; easy to use; familiar technology to many women and those assigned female at birth.
**Pad**	**Q-Pad™** Company: Qvin (formerly Qurasense) City: Menlo Park, CA Country: USA Introduced 2022–2024. Commercialised in the USA.	Modified menstrual pad designed for passive high-risk HPV (hrHPV) sample collection. It includes a removable collection strip that processes specimens as dried blood spots.	Sensitivity: 82.8–97.7% [137]. Specificity: 50–98.0% [137].		100% [136].	29 USD.	Familiar technology to many women and those assigned female at birth. Self-collection only possible during menstruation.
**Urine Assay**	**Self UriSponge™** Company: COPAN Group Headquarters: Brescia, Lombardy, Italy US Operations: Murrieta, CA, USA Introduced 2024. Globally commercialised.	Plastic tube with polyurethane sponge, saturated with boric acid and sodium formate as a transportation and storage medium.					Non-invasive; easy to use. Sample can be stored up to 48 h at 25 °C.
	**Colli-Pee^®^** Company: DNA Genotek (subsidiary of OraSure Technologies, Inc.) City: Ottawa, ON Country: Canada Note: Novosanis, the original developer, is now part of DNA Genotek. Introduced in 2016. Commercialised in the USA, UK and Europe.	First-Void Urine (FVU) device consisting of a plastic sample tube, with a funnel collector tube attached.	Sensitivity 89% (75–97%) [139]. Specificity: 98% (95–99%) [139].	Sensitivity: 90.9% (82.4–99.4%) [113]. Specificity: 39.8% (3.0–46.6%) [113].	Concordance rates between cervical and urine specimens were 90.6% (with k = 0.792) for hr-HPV and 85.7% (with k = 0.715) for lr-HPV [113].	2.58–5.18 EUR; easy to use [112]; acceptable [138].	Non-invasive; easy to use.

### 5.1. Device Types and Clinical Performance

The Evalyn^®^ Brush (Rovers Medical Devices) and FLOQSwabs™ (COPAN Group) represent extensively validated self-sampling methods, with the Evalyn^®^ Brush achieving 97% user acceptability through innovative features like depth indicators and click mechanisms [118,141]. FLOQSwabs™ use short nylon fibres arranged on a solid plastic shaft. The fibres are positioned perpendicularly to promote strong capillary action, allowing for the rapid absorption of liquid samples. As it lacks an internal core, more than 90% of the collected sample can be easily released into testing media. While urine-based testing offers minimal invasiveness and potential integration with other screening programmes, it demonstrates lower sensitivity (51–63%) compared to other self-sampling methods, limiting its current utility as a primary screening approach [142].

The Daye Diagnostic Tampon (DT) shows promising performance with 82.9% sensitivity and 91.6% specificity, achieving the highest valid result rates (99.2%) compared to vaginal self-swabs (95.4%) and clinician-collected samples (90.8%) [135]. A valid result refers to the proportion of samples collected using the DT that were deemed adequate and suitable for laboratory analysis, meaning they met the necessary quality criteria for accurate HPV testing. User acceptance is high, with 78.3% reporting high comfort pre-sampling and 74.5% finding it “very easy” to use [135,143]. When collected first in the sampling sequence, the DT achieves optimal performance (100% sensitivity, 96.8% specificity), with 70.5% of participants preferring this method [135].

Teal Health was granted Breakthrough Device Designation by the FDA in 2024 for their Teal Wand device [144]. It has integrated digital features, including visualisation capabilities and automated sample verification, achieving 94% sample adequacy rates and higher user satisfaction [131]. The Papcup system is a novel biosensor point-of-care device that enables rapid HPV detection from menstrual blood samples, delivering results within 15 min. This innovative approach enhances accessibility in low-resource settings by eliminating the need for complex laboratory infrastructure while offering a non-invasive and culturally acceptable screening option for underserved populations [145]. A novel approach using a modified menstrual pad (Q-Pad) for passive HPV sample collection shows high concordance (95–100%) with clinician-collected samples among HPV-positive women, offering potential for integration into cervical cancer prevention programmes [136].

A collection of studies examined the effectiveness of self-sampling methods for HPV testing compared to clinician-taken cervical samples. Vaginal self-sampling using dry flocked swabs, wet Dacron swabs and urine samples showed similar sensitivity and specificity to clinician-taken samples for detecting high-grade cervical lesions [92,146,147]. Self-sampling methods were generally well-accepted by women, with urine collection being the easiest and most preferred option [92].

Digital health solutions demonstrate significant benefits in cervical cancer screening programmes, with electronic health interventions improving screening participation rates by 46% compared to usual care, with a particularly strong impact in LMIC settings [148]. Malaysia’s Program ROSE integrates self-sampling, primary HPV testing and a digital health registry to ensure timely communication of results and linkage to care. This initiative has screened over 25,000 women, achieving high acceptability rates and empowering underserved populations through mobile technology [149]. However, a critical limitation is the requirement for additional cytology visits following HPV-positive results, leading to 35–45% patient dropout rates during follow-up [150]. While newer devices attempt dual sample collection for both HPV and cytology testing, cytological examination from self-collected samples shows lower adequacy rates compared to clinician collection, primarily due to their inability to sample the cervical transformation zone where precancerous lesions typically originate [151]. Current self-sampling devices face limitations in accessing endocervical cells within the transformation zone, a critical area for identifying precancerous changes, requiring speculum-based clinician collection for comprehensive cervical screening [140,152]. Despite this drawback, self-sampling is proposed as a complementary strategy to increase screening participation, particularly among underserved populations who face barriers to clinic-based care, such as discomfort, embarrassment, or logistical challenges.

Self-sampling technologies, including novel approaches like the Diagnostic Tampon, and the Teal Wand, show promise for increasing screening accessibility. Meta-analyses demonstrate that self-sampling can increase participation by 1.5 to 2.5 times compared to traditional methods, particularly impacting underserved populations [153,154].

The integration of AI and molecular testing enhances screening precision, with AI algorithms demonstrating 95% accuracy in identifying cervical abnormalities [155]. POC testing, showing 93% sensitivity and 91% specificity, offers potential solutions for resource-limited settings [156].

### 5.2. Advancements in DNA Methylation Testing

Recent advancements in DNA methylation testing represent a significant breakthrough in cervical cancer screening strategies. Meta-analyses demonstrate that DNA methylation markers achieve 63% sensitivity and 76% specificity for CIN2+ and 71% sensitivity and 75% specificity for CIN3+ [157]. This approach effectively identifies women at higher risk of progression to cancer while reducing unnecessary referrals. The WID-qCIN test, which assesses the methylation of DPP6, RALYL and GSX1 genes, demonstrated improved performance over cytology in a large real-world cohort [158]. While methylation assays initially require higher investment in molecular infrastructure, their superior sensitivity (63–71% for CIN2+/CIN3+) and reduced need for specialised cytology expertise make them potentially cost-effective for LMICs in the long term [159]. There is the potential to repurpose COVID testing PCR equipment, which is universally available, reducing investment costs in DNA methylation. Self-sampling in combination with DNA methylation testing presents a promising pathway for cervical cancer screening innovation, potentially streamlining the screening process by eliminating separate cytology testing and reducing reliance on clinical infrastructure while maintaining high diagnostic standards.

HPV genotyping provides another effective triage strategy, already implemented in countries like the Netherlands. This approach allows for risk stratification of HPV-positive self-samples based on type-specific risk, though implementation in LMICs requires consideration of cost and laboratory infrastructure. Studies show that genotyping can effectively identify women requiring immediate colposcopy versus those suitable for routine screening intervals [160,161].

Successful implementation requires the careful consideration of quality assurance, healthcare integration and cost analysis. This innovative combination of self-sampling, methylation testing and POC analysis represents a potentially transformative approach to cervical cancer screening, aligned with WHO’s elimination goals, and could significantly improve screening accessibility and effectiveness in low-resource settings while maintaining high diagnostic standards.

## 6. Conclusions

The landscape of cervical cancer prevention is transforming through emerging technologies and evolving healthcare strategies. While progress has been made, substantial disparities persist between HICs and LMICs, necessitating innovative solutions. 

Cost-effectiveness analyses indicate that integrated digital health solutions can reduce screening costs by 46% in low-resource settings while improving follow-up rates. However, successful implementation requires careful consideration of quality assurance, follow-up pathways, healthcare infrastructure integration and cultural acceptability.

As HPV vaccination coverage increases globally, screening protocols will require adjustment, with evidence suggesting extended intervals for vaccinated populations [58]. Critical knowledge gaps require focused research, including standardised quality metrics for sample adequacy and evidence-based risk-stratification algorithms. Research priorities vary by healthcare setting, with HICs focusing on AI integration and multi-cancer detection platforms, while LMICs require cost-effective sample transport systems and POC testing validation.

The goal of cervical cancer elimination appears increasingly achievable through these innovative approaches, but success depends on addressing implementation challenges, particularly in resource-limited settings.

## Data Availability

Not applicable.

## References

[1] Chandra Sekar P.K., Thomas S.M., Veerabathiran R. (2024). The future of cervical cancer prevention: Advances in research and technology. Explor. Med..

[2] Ramanujam N. Accelerating the impact of technology and innovation for global cervical cancer prevention. Proceedings of the SPIE BIOS.

[3] Cox J.T. (2006). Epidemiology and natural history of HPV. J. Fam. Pract..

[4] Jensen J.E., Becker G.L., Jackson J.B., Rysavy M.B. (2024). Human Papillomavirus and Associated Cancers: A Review. Viruses.

[5] Ong S.K., Abe S.K., Thilagaratnam S., Haruyama R., Pathak R., Jayasekara H., Togawa K., Bhandari A.K.C., Shankar A., Nessa A. (2023). Towards elimination of cervical cancer—human papillomavirus (HPV) vaccination and cervical cancer screening in Asian National Cancer Centers Alliance (ANCCA) member countries. Lancet Reg. Health West. Pac..

[6] Okunade K.S. (2020). Human papillomavirus and cervical cancer. J. Obstet. Gynaecol..

[7] Singh D., Vignat J., Lorenzoni V., Eslahi M., Ginsburg O., Lauby-Secretan B., Arbyn M., Basu P., Bray F., Vaccarella S. (2022). Global estimates of incidence and mortality of cervical cancer in 2020: A baseline analysis of the WHO Global Cervical Cancer Elimination Initiative. Lancet Glob. Health.

[8] Viveros-Carreño D., Fernandes A., Pareja R. (2023). Updates on cervical cancer prevention. Int. J. Gynecol. Cancer.

[9] WHO Cervical Cancer Elimination Initiative. https://www.who.int/initiatives/cervical-cancer-elimination-initiative.

[10] Vaccarella S., Laversanne M., Ferlay J., Bray F. (2017). Cervical cancer in Africa, Latin America and the Caribbean and Asia: Regional inequalities and changing trends. Int. J. Cancer.

[11] Hull R., Mbele M., Makhafola T., Hicks C., Wang S.-M., Reis R.M., Mehrotra R., Mkhize-Kwitshana Z., Kibiki G., Bates D.O. (2020). Cervical cancer in low and middle-income countries. Oncol. Lett..

[12] WHO Cervical Cancer. https://www.who.int/news-room/fact-sheets/detail/cervical-cancer.

[13] Spencer J.C., Brewer N.T., Coyne-Beasley T., Trogdon J.G., Weinberger M., Wheeler S.B. (2021). Reducing Poverty-related Disparities in Cervical Cancer: The Role of HPV Vaccination. Cancer Epidemiol. Biomark. Prev. Publ. Am. Assoc. Cancer Res. Cosponsored Am. Soc. Prev. Oncol..

[14] Falcaro M., Soldan K., Ndlela B., Sasieni P. (2024). Effect of the HPV vaccination programme on incidence of cervical cancer and grade 3 cervical intraepithelial neoplasia by socioeconomic deprivation in England: Population based observational study. BMJ.

[15] NHS Cervical Screening Programme Audit of Invasive Cervical Cancer: National Report 1 April 2016 to 31 March 2019. https://www.gov.uk/government/publications/cervical-screening-invasive-cervical-cancer-audit-2016-to-2019/nhs-cervical-screening-programme-audit-of-invasive-cervical-cancer-national-report-1-april-2016-to-31-march-2019.

[16] Choi S., Ismail A., Pappas-Gogos G., Boussios S. (2023). HPV and Cervical Cancer: A Review of Epidemiology and Screening Uptake in the UK. Pathogens.

[17] Hira R.K., Akomfrah G. (2024). Ealing.gov.uk Cancer Screening—Uptake, Diagnosis and Inequalities in Ealing. https://ealing.moderngov.co.uk/documents/s14383/Appendix+1+Cancer+screening+Uptake+diagnosis+and+inequalities.pdf.

[18] Cervical Screening Standards Data Report 2022 to 2023. https://www.gov.uk/government/publications/cervical-screening-standards-data-report-2022-to-2023/cervical-screening-standards-data-report-2022-to-2023.

[19] NHS England NHS England NHS Sets Ambition to Eliminate Cervical Cancer by 2040. https://www.england.nhs.uk/2023/11/nhs-sets-ambition-to-eliminate-cervical-cancer-by-2040/.

[20] Kundrod K.A., Jeronimo J., Vetter B., Maza M., Murenzi G., Phoolcharoen N., Castle P.E. (2023). Toward 70% cervical cancer screening coverage: Technical challenges and opportunities to increase access to human papillomavirus (HPV) testing. PLOS Glob. Public Health.

[21] Gravitt P.E., Silver M.I., Hussey H.M., Arrossi S., Huchko M., Jeronimo J., Kapambwe S., Kumar S., Meza G., Nervi L. (2021). Achieving equity in cervical cancer screening in low- and middle-income countries (LMICs): Strengthening health systems using a systems thinking approach. Prev. Med..

[22] eClinicalMedicine (2023). Global strategy to eliminate cervical cancer as a public health problem: Are we on track?. eClinicalMedicine.

[23] Allanson E.R., Schmeler K.M. (2021). Preventing Cervical Cancer Globally: Are We Making Progress?. Cancer Prev. Res..

[24] Canfell K. (2019). Towards the global elimination of cervical cancer. Papillomavirus Res..

[25] Palmer T.J., Kavanagh K., Cuschieri K., Cameron R., Graham C., Wilson A., Roy K. (2024). Invasive cervical cancer incidence following bivalent human papillomavirus vaccination: A population-based observational study of age at immunization, dose, and deprivation. J. Natl. Cancer Inst..

[26] Hall M.T., Simms K.T., Lew J.-B., Smith M.A., Saville M., Canfell K. (2018). Projected future impact of HPV vaccination and primary HPV screening on cervical cancer rates from 2017–2035: Example from Australia. PLoS ONE.

[27] Luckett R., Feldman S. (2016). Impact of 2-, 4- and 9-valent HPV vaccines on morbidity and mortality from cervical cancer. Hum. Vaccines Immunother..

[28] Maver P.J., Poljak M. (2020). Primary HPV-based cervical cancer screening in Europe: Implementation status, challenges, and future plans. Clin. Microbiol. Infect..

[29] Wentzensen N., Arbyn M. (2017). HPV-based cervical cancer screening- facts, fiction, and misperceptions. Prev. Med..

[30] Lei J., Ploner A., Elfström K.M., Wang J., Roth A., Fang F., Sundström K., Dillner J., Sparén P. (2020). HPV Vaccination and the Risk of Invasive Cervical Cancer. N. Engl. J. Med..

[31] Orumaa M., Lahlum E.J., Gulla M., Tota J.E., Nygård M., Nygård S. (2024). Quadrivalent HPV Vaccine Effectiveness Against Cervical Intraepithelial Lesion Grade 2 or Worse in Norway: A Registry-Based Study of 0.9 Million Norwegian Women. J. Infect. Dis..

[32] Mikalsen M.P., Simonsen G.S., Sørbye S.W. (2024). Impact of HPV Vaccination on the Incidence of High-Grade Cervical Intraepithelial Neoplasia (CIN2+) in Women Aged 20–25 in the Northern Part of Norway: A 15-Year Study. Vaccines.

[33] Ellingson M.K., Sheikha H., Nyhan K., Oliveira C.R., Niccolai L.M. (2023). Human papillomavirus vaccine effectiveness by age at vaccination: A systematic review. Hum. Vaccines Immunother..

[34] Tsu V.D., LaMontagne D.S., Atuhebwe P., Bloem P.N., Ndiaye C. (2021). National implementation of HPV vaccination programs in low-resource countries: Lessons, challenges, and future prospects. Prev. Med..

[35] Guignard A., Praet N., Jusot V., Bakker M., Baril L. (2019). Introducing new vaccines in low- and middle-income countries: Challenges and approaches. Expert Rev. Vaccines.

[36] Kumar S., Butler D. (2013). Calls in India for legal action against US charity. Nature.

[37] Swanson A.A., Pantanowitz L. (2024). The evolution of cervical cancer screening. J. Am. Soc. Cytopathol..

[38] Chatterjee P.B., Hingway S.R., Hiwale K.M. (2024). Evolution of Pathological Techniques for the Screening of Cervical Cancer: A Comprehensive Review. Cureus.

[39] Sharma J., Yennapu M., Priyanka Y. (2023). Screening Guidelines and Programs for Cervical Cancer Control in Countries of Different Economic Groups: A Narrative Review. Cureus.

[40] Arbyn M., Ronco G., Anttila A., Meijer C.J.L.M., Poljak M., Ogilvie G., Koliopoulos G., Naucler P., Sankaranarayanan R., Peto J. (2012). Evidence Regarding Human Papillomavirus Testing in Secondary Prevention of Cervical Cancer. Vaccine.

[41] Ogilvie G., Nakisige C., Huh W.K., Mehrotra R., Franco E.L., Jeronimo J. (2017). Optimizing secondary prevention of cervical cancer: Recent advances and future challenges. Int. J. Gynecol. Obstet..

[42] WHO Recommends DNA Testing as a First-Choice Screening Method for Cervical Cancer Prevention. https://www.who.int/europe/news-room/11-09-2021-who-recommends-dna-testing-as-a-first-choice-screening-method-for-cervical-cancer-prevention.

[43] Murewanhema G., Dzobo M., Moyo E., Moyo P., Mhizha T., Dzinamarira T. (2023). Implementing HPV-DNA screening as primary cervical cancer screening modality in Zimbabwe: Challenges and recommendations. Sci. Afr..

[44] Lozar T., Nagvekar R., Rohrer C., Dube Mandishora R.S., Ivanus U., Fitzpatrick M.B. (2021). Cervical Cancer Screening Postpandemic: Self-Sampling Opportunities to Accelerate the Elimination of Cervical Cancer. Int. J. Womens Health.

[45] Serrano B., Ibáñez R., Robles C., Peremiquel-Trillas P., De Sanjosé S., Bruni L. (2022). Worldwide use of HPV self-sampling for cervical cancer screening. Prev. Med..

[46] Chan C.K., Aimagambetova G., Ukybassova T., Kongrtay K., Azizan A. (2019). Human Papillomavirus Infection and Cervical Cancer: Epidemiology, Screening, and Vaccination—Review of Current Perspectives. J. Oncol..

[47] Eun T.J., Perkins R.B. (2020). Screening for Cervical Cancer. Med. Clin. N. Am..

[48] Cervical Cancer Causes, Risk Factors, and Prevention—NCI. https://www.cancer.gov/types/cervical/causes-risk-prevention.

[49] Lowy D.R., Solomon D., Hildesheim A., Schiller J.T., Schiffman M. (2008). Human papillomavirus infection and the primary and secondary prevention of cervical cancer. Cancer.

[50] Bruni L., Diaz M., Barrionuevo-Rosas L., Herrero R., Bray F., Bosch F.X., de Sanjosé S., Castellsagué X. (2016). Global estimates of human papillomavirus vaccination coverage by region and income level: A pooled analysis. Lancet Glob. Health.

[51] (2017). References Human Papillomavirus Vaccines: WHO Position Paper, May 2017 (References with Abstracts Cited in the Position Paper in the Order of Appearance). SAGE Guidance for the Development of Evidence-Based Vaccine-Related Recommendations. https://www.semanticscholar.org/paper/References-Human-papillomavirus-vaccines%3A-WHO-May/7dc6c6b55657f911c80ea0427208cb1ff5aa913c.

[52] Chen J.J. (2010). Genomic instability induced by human papillomavirus oncogenes. N. Am. J. Med. Sci..

[53] Korzeniewski N., Spardy N., Duensing A., Duensing S. (2011). Genomic instability and cancer: Lessons learned from human papillomaviruses. Cancer Lett..

[54] Balasubramaniam S.D., Balakrishnan V., Oon C.E., Kaur G. (2019). Key Molecular Events in Cervical Cancer Development. Medicina.

[55] Mello V., Sundstrom R.K. (2019). Cervical Intraepithelial Neoplasia. StatPearls.

[56] Cooper D.B., Dunton C.J. (2021). Colposcopy. StatPearls.

[57] Arbyn M., Xu L. (2018). Efficacy and safety of prophylactic HPV vaccines. A Cochrane review of randomized trials. Expert Rev. Vaccines.

[58] Giorgi Rossi P., Carozzi F., Federici A., Ronco G., Zappa M., Franceschi S., Barca A., Barzon L., Baussano I., Berliri C. (2017). Cervical cancer screening in women vaccinated against human papillomavirus infection: Recommendations from a consensus conference. Prev. Med..

[59] Zhai L., Tumban E. (2016). Gardasil-9: A global survey of projected efficacy. Antiviral Res..

[60] Signorelli C., Odone A., Ciorba V., Cella P., Audisio R.A., Lombardi A., Mariani L., Mennini F.S., Pecorelli S., Rezza G. (2017). Human papillomavirus 9-valent vaccine for cancer prevention: A systematic review of the available evidence. Epidemiol. Infect..

[61] Silver M.I., Kobrin S. (2020). Exacerbating disparities?: Cervical cancer screening and HPV vaccination. Prev. Med..

[62] Staley H., Shiraz A., Shreeve N., Bryant A., Martin-Hirsch P.P., Gajjar K. (2021). Interventions targeted at women to encourage the uptake of cervical screening. Cochrane Database Syst. Rev..

[63] Stanley M., Schuind A., Muralidharan K.K., Guillaume D., Willens V., Borda H., Jurgensmeyer M., Limaye R. (2024). Evidence for an HPV one-dose schedule. Vaccine.

[64] NCI PRIMAVERA Immunobridging Trial. https://dceg.cancer.gov/research/cancer-types/cervix/primavera.

[65] D’Amato S., Nunnari G., Trimarchi G., Squeri A., Cancellieri A., Squeri R., Pellicanò G.F. (2022). Impact of the COVID-19 pandemic on HPV vaccination coverage in the general population and in PLWHs. Eur. Rev. Med. Pharmacol. Sci..

[66] Castanon A., Rebolj M., Pesola F., Pearmain P., Stubbs R. (2022). COVID-19 disruption to cervical cancer screening in England. J. Med. Screen..

[67] Ramírez A.T., Valls J., Baena A., Rojas F.D., Ramírez K., Álvarez R., Cristaldo C., Henríquez O., Moreno A., Reynaga D.C. (2023). Performance of cervical cytology and HPV testing for primary cervical cancer screening in Latin America: An analysis within the ESTAMPA study. Lancet Reg. Health Am..

[68] Cuschieri K., Wentzensen N. (2008). Human Papillomavirus mRNA and p16 Detection as Biomarkers for the Improved Diagnosis of Cervical Neoplasia. Cancer Epidemiol. Biomarkers Prev..

[69] Cuschieri K., Ronco G., Lorincz A., Smith L., Ogilvie G., Mirabello L., Carozzi F., Cubie H., Wentzensen N., Snijders P. (2018). Eurogin roadmap 2017: Triage strategies for the management of HPV-positive women in cervical screening programs. Int. J. Cancer.

[70] Hall M.T., Simms K.T., Lew J.-B., Smith M.A., Brotherton J.M., Saville M., Frazer I.H., Canfell K. (2019). The projected timeframe until cervical cancer elimination in Australia: A modelling study. Lancet Public Health.

[71] Bryant E. (2012). The impact of policy and screening on cervical cancer in England. Br. J. Nurs..

[72] Arbyn M., Costa S., Latsuzbaia A., Kellen E., Girogi Rossi P., Cocuzza C.E., Basu P., Castle P.E. (2023). HPV-based Cervical Cancer Screening on Self-samples in the Netherlands: Challenges to Reach Women and Test Performance Questions. Cancer Epidemiol. Biomarkers Prev..

[73] Elfström M., Gray P.G., Dillner J. (2023). Cervical cancer screening improvements with self-sampling during the COVID-19 pandemic. eLife.

[74] Wirtz C., Mohamed Y., Engel D., Sidibe A., Holloway M., Bloem P., Kumar S., Brotherton J., Reis V., Morgan C. (2022). Integrating HPV vaccination programs with enhanced cervical cancer screening and treatment, a systematic review. Vaccine.

[75] Ebrahimi N., Yousefi Z., Khosravi G., Malayeri F.E., Golabi M., Askarzadeh M., Shams M.H., Ghezelbash B., Eskandari N. (2023). Human papillomavirus vaccination in low- and middle-income countries: Progression, barriers, and future prospective. Front. Immunol..

[76] Sankaranarayanan R., Qiao Y., Keita N. (2015). The Next Steps in Cervical Screening. Womens Health.

[77] Binagwaho A., Wagner C., Gatera M., Karema C., Nutt C., Ngaboa F. (2012). Achieving high coverage in Rwanda’s national human papillomavirus vaccination programme. Bull. World Health Organ..

[78] Poli U.R., Muwonge R., Bhoopal T., Lucas E., Basu P. (2020). Feasibility, Acceptability, and Efficacy of a Community Health Worker–Driven Approach to Screen Hard-to-Reach Periurban Women Using Self-Sampled HPV Detection Test in India. JCO Glob. Oncol..

[79] WHO (2022). Tracking Universal Health Coverage in the WHO African Region. https://www.afro.who.int/publications/tracking-universal-health-coverage-who-african-region-2022.

[80] Okolie E.A., Aluga D., Anjorin S., Ike F.N., Ani E.M., Nwadike B.I. (2022). Addressing missed opportunities for cervical cancer screening in Nigeria: A nursing workforce approach. Ecancermedicalscience.

[81] WHO WHO Cervical Cancer Elimination Initiative: From Call to Action to Global Movement. https://www.who.int/publications/m/item/who-cervical-cancer-elimination-initiative--from-call-to-action-to-global-movement.

[82] Goldhaber-Fiebert J.D., Stout N.K., Salomon J.A., Kuntz K.M., Goldie S.J. (2008). Cost-Effectiveness of Cervical Cancer Screening With Human Papillomavirus DNA Testing and HPV-16,18 Vaccination. JNCI J. Natl. Cancer Inst..

[83] WHO Zambia Steps up Cervical Cancer Screening with HPV Testing WHO Regional Office for Africa. https://www.afro.who.int/countries/zambia/news/zambia-steps-cervical-cancer-screening-hpv-testing.

[84] Desta A.A., Alemu F.T., Gudeta M.B., Dirirsa D.E., Kebede A.G. (2022). Willingness to utilize cervical cancer screening among Ethiopian women aged 30–65 years. Front. Glob. Womens Health.

[85] Clinton Health Access Initiative Scaling Up an Effective Model of Care to Prevent and Treat Cervical Cancer in Rwanda. https://www.clintonhealthaccess.org/blog/scaling-up-an-effective-model-of-care-to-prevent-and-treat-cervical-cancer-in-rwanda/.

[86] Wave of New Commitments Marks Historic Step Towards the Elimination of Cervical Cancer. https://www.gavi.org/news/media-room/wave-new-commitments-marks-historic-step-towards-elimination-cervical-cancer.

[87] Toliman P.J., Kaldor J.M., Tabrizi S.N., Vallely A.J. (2018). Innovative approaches to cervical cancer screening in low- and middle-income countries. Climacteric.

[88] Davies-Oliveira J.C., Smith M.A., Grover S., Canfell K., Crosbie E.J. (2021). Eliminating Cervical Cancer: Progress and Challenges for High-income Countries. Clin. Oncol..

[89] Ibrahim A., Simeen N. (2024). 32P Cervical cancer: Barriers and smears to prevention. ESMO Open.

[90] Waller J., Bartoszek M., Marlow L., Wardle J. (2009). Barriers to cervical cancer screening attendance in England: A population-based survey. J. Med. Screen..

[91] Marlow L., McBride E., Varnes L., Waller J. (2019). Barriers to cervical screening among older women from hard-to-reach groups: A qualitative study in England. BMC Womens Health.

[92] Cadman L., Waller J., Ashdown-Barr L., Szarewski A. (2012). Barriers to cervical screening in women who have experienced sexual abuse: An exploratory study: Table 1. J. Fam. Plann. Reprod. Health Care.

[93] Berner A.M., Connolly D.J., Pinnell I., Wolton A., MacNaughton A., Challen C., Nambiar K., Bayliss J., Barrett J., Richards C. (2021). Attitudes of transgender men and non-binary people to cervical screening: A cross-sectional mixed-methods study in the UK. Br. J. Gen. Pract. J. R. Coll. Gen. Pract..

[94] Dhillon N., Oliffe J.L., Kelly M.T., Krist J. (2020). Bridging Barriers to Cervical Cancer Screening in Transgender Men: A Scoping Review. Am. J. Men’s Health.

[95] Lim A.W., Deats K., Gambell J., Lawrence A., Lei J., Lyons M., North B., Parmar D., Patel H., Waller J. (2024). Opportunistic offering of self-sampling to non-attenders within the English cervical screening programme: A pragmatic, multicentre, implementation feasibility trial with randomly allocated cluster intervention start dates (YouScreen). eClinicalMedicine.

[96] Mathews C., Brentnall A., Rebolj M., Sargent A., Cuschieri K., Denton K. (2024). HPValidate: Clinical Validation of hrHPV Test System Using Self-Collected Vaginal Samples in NHS England Commissioned Laboratories Providing Cervical Screening Services Core Reporting Group. https://www.qmul.ac.uk/fmd/media/smd/documents/research/hpv-self-collection-test-accuracy-report-hpvalidate-lot1.pdf.

[97] Hariprasad R., John A., Abdulkader R.S. (2023). Challenges in the Implementation of Human Papillomavirus Self-Sampling for Cervical Cancer Screening in India: A Systematic Review. JCO Glob. Oncol..

[98] Woo Y.L., Gravitt P., Khor S.K., Ng C.W., Saville M. (2021). Accelerating action on cervical screening in lower- and middle-income countries (LMICs) post COVID-19 era. Prev. Med..

[99] WHO Feasibility of the WHO Strategy to Eliminate Cervical Cancer as a Public Health Problem, Lessons Learned from the PRESCRIP-TEC Project Knowledge Action Portal on NCDs. https://www.knowledge-action-portal.com/en/content/feasibility-who-strategy-eliminate-cervical-cancer-public-health-problem-lessons-learned.

[100] Gupta S., Palmer C., Bik E.M., Cardenas J.P., Nuñez H., Kraal L., Bird S.W., Bowers J., Smith A., Walton N.A. (2018). Self-Sampling for Human Papillomavirus Testing: Increased Cervical Cancer Screening Participation and Incorporation in International Screening Programs. Front. Public Health.

[101] Arbyn M., Smith S.B., Temin S., Sultana F., Castle P. (2018). Detecting cervical precancer and reaching underscreened women by using HPV testing on self samples: Updated meta-analyses. BMJ.

[102] Viñals R., Jonnalagedda M., Petignat P., Thiran J.-P., Vassilakos P. (2023). Artificial Intelligence-Based Cervical Cancer Screening on Images Taken during Visual Inspection with Acetic Acid: A Systematic Review. Diagnostics.

[103] Gupta R., Sarwar A., Sharma V. (2017). Screening of Cervical Cancer by Artificial Intelligence based Analysis of Digitized Papani-colaou-Smear Images. https://www.semanticscholar.org/paper/Screening-of-Cervical-Cancer-by-Artificial-based-of-Gupta-Sarwar/44acfd2e0a27500a100cf31ad9c9f5d018997bac.

[104] Wu T., Lucas E., Zhao F., Basu P., Qiao Y. (2024). Artificial intelligence strengthenes cervical cancer screening—Present and future. Cancer Biol. Med..

[105] Gupta R., Gupta S. (2023). Point-of-care tests for human papillomavirus detection in uterine cervical samples: A review of advances in resource-constrained settings. Indian J. Med Res..

[106] Seely S., Zingg J.-M., Joshi P., Slomovitz B., Schlumbrecht M., Kobetz E., Deo S., Daunert S. (2023). Point-of-Care Molecular Test for the Detection of 14 High-Risk Genotypes of Human Papillomavirus in a Single Tube. Anal. Chem..

[107] Kelly H., Mayaud P., Segondy M., Pai N.P., Peeling R.W. (2017). A systematic review and meta-analysis of studies evaluating the performance of point-of-care tests for human papillomavirus screening. Sex. Transm. Infect..

[108] Kuhn L., Denny L. (2017). The time is now to implement HPV testing for primary screening in low resource settings. Prev. Med..

[109] Vallely A.J.B., Saville M., Badman S.G., Gabuzzi J., Bolnga J., Mola G.D.L., Kuk J., Wai M., Munnull G., Garland S.M. (2022). Point-of-care HPV DNA testing of self-collected specimens and same-day thermal ablation for the early detection and treatment of cervical pre-cancer in women in Papua New Guinea: A prospective, single-arm intervention trial (HPV-STAT). Lancet Glob. Health.

[110] Shrivas S., Patel M., Kumar R., Gwal A., Uikey R., Tiwari S.K., Verma A.K., Thota P., Das A., Bharti P.K. (2021). Evaluation of Microchip-Based Point-Of-Care Device “Gazelle” for Diagnosis of Sickle Cell Disease in India. Front. Med..

[111] WHO Target Product Profiles for Human Papillomavirus Screening Tests to Detect Cervical Pre-Cancer and Cancer. https://www.who.int/publications/i/item/9789240100275.

[112] Cadman L., Reuter C., Jitlal M., Kleeman M., Austin J., Hollingworth T., Parberry A.L., Ashdown-Barr L., Patel D., Nedjai B. (2021). A Randomized Comparison of Different Vaginal Self-sampling Devices and Urine for Human Papillomavirus Testing—Predictors 5.1. Cancer Epidemiol. Biomark. Prev..

[113] Martinelli M., Giubbi C., Di Meo M.L., Perdoni F., Musumeci R., Leone B.E., Fruscio R., Landoni F., Cocuzza C.E. (2023). Accuracy of Human Papillomavirus (HPV) Testing on Urine and Vaginal Self-Samples Compared to Clinician-Collected Cervical Sample in Women Referred to Colposcopy. Viruses.

[114] Ertik F.C., Kampers J., Hülse F., Stolte C., Böhmer G., Hillemanns P., Jentschke M. (2021). CoCoss-Trial: Concurrent Comparison of Self-Sampling Devices for HPV-Detection. Int. J. Environ. Res. Public Health.

[115] Sechi I., Muresu N., Puci M.V., Saderi L., Del Rio A., Cossu A., Muroni M.R., Castriciano S., Martinelli M., Cocuzza C.E. (2023). Preliminary Results of Feasibility and Acceptability of Self-Collection for Cervical Screening in Italian Women. Pathogens.

[116] Saville M., Hawkes D., Keung M., Ip E., Silvers J., Sultana F., Malloy M.J., Velentzis L.S., Canfel L.K., Wrede C.D. (2020). Analytical performance of HPV assays on vaginal self-collected vs practitioner-collected cervical samples: The SCoPE study. J. Clin. Virol..

[117] Hon H.J., Chong P.P., Choo H.L., Khine P.P. (2023). A Comprehensive Review of Cervical Cancer Screening Devices: The Pros and the Cons. Asian Pac. J. Cancer Prev..

[118] Leinonen M.K., Schee K., Jonassen C.M., Lie A.K., Nystrand C.F., Rangberg A., Furre I.E., Johansson M.J., Tropé A., Sjøborg K.D. (2018). Safety and acceptability of human papillo-mavirus testing of self-collected specimens: A methodologic study of the impact of collection devices and HPV assays on sensitivity for cervical cancer and high-grade lesions. J. Clin Virol..

[119] Bokan T., Ivanus U., Jerman T., Takac I., Arko D. (2021). Long term results of follow-up after HPV self-sampling with devices Qvintip and HerSwab in women non-attending cervical screening programme. Radiol. Oncol..

[120] Latsuzbaia A., Van Keer S., Broeck D.V., Weyers S., Donders G., De Sutter P., Tjalma W., Doyen J., Vorsters A., Arbyn M. (2023). Clinical Accuracy of Alinity m HR HPV Assay on Self- versus Clinician-Taken Samples Using the VALHUDES Protocol. J. Mol. Diagn..

[121] Jentschke M., Chen K., Arbyn M., Hertel B., Noskowicz M., Soergel P., Hillemanns P. (2016). Direct comparison of two vaginal self-sampling devices for the detection of human papillomavirus infections. J. Clin. Virol..

[122] El-Zein M., Bouten S., Louvanto K., Gilbert L., Gotlieb W., Hemmings R., Behr M.A., Franco E.L. (2018). Validation of a new HPV self-sampling device for cervical cancer screening: The Cervical and Self-Sample In Screening (CASSIS) study. Gynecol. Oncol..

[123] Chernesky M., Hook E.W., Martin D.H., Lane J., Johnson R., Jordan J.A., Fuller D., Willis D.E., Fine P.M., Janda W.M. (2005). Women find it easy and prefer to collect their own vaginal swabs to diagnose Chlamydia trachomatis or Neisseria gonorrhoeae infections. Sex. Transm. Dis..

[124] CADTH (Agency for Drugs and Technologies in Health) (2017). Health Technology Update: Issue 18. https://www.cda-amc.ca/sites/default/files/pdf/Health_Technology_Update_Issue_18.pdf.

[125] Polman N.J., Ebisch R.M.F., Heideman D.A.M., Melchers W.J.G., Bekkers R.L.M., Molijn A.C., Meijer C.J.L.M., Quint W.G.V., Snijders P.J.F., Massuger L.F.A.G. (2019). Performance of human papillomavirus testing on self-collected versus clinician-collected samples for the detection of cervical intraepithelial neoplasia of grade 2 or worse: A randomised, paired screen-positive, non-inferiority trial. Lancet Oncol..

[126] van Baars R., Bosgraaf R.P., ter Harmsel B.W.A., Melchers W.J.G., Quint W.G.V., Bekkers R.L.M. (2012). Dry storage and transport of a cer-vicovaginal self-sample by use of the Evalyn Brush, providing reliable human papillomavirus detection combined with comfort for women. J. Clin. Microbiol..

[127] Ketelaars P.J.W., Bosgraaf R.P., Siebers A.G., Massuger L.F.A.G., van der Linden J.C., Wauters C.A.P., Rahamat-Langendoen J.C., van den Brule A.J.C., IntHout J., Melchers W.J.G. (2017). High-risk human papillomavirus detection in self-sampling compared to physician-taken smear in a responder population of the Dutch cervical screening: Results of the VERA study. Prev. Med..

[128] Bosgraaf R.P., Verhoef V.M.J., Massuger L.F.A.G., Siebers A.G., Bulten J., de Kuyper-de Ridder G.M., Meijer C.J.M., Snijders P.J.F., Heideman D.A.M., IntHout J. (2015). Comparative performance of novel self-sampling methods in detecting high-risk human papillomavirus in 30,130 women not attending cervical screening. Int. J. Cancer.

[129] Okayama K., Okodo M., Fujii M., Kumagai T., Yabusaki H., Shiina Y., Iwami F., Teruya K., Hatta K. (2012). Improved accuracy of cytodiagnosis using the Kato self-collection devise: The usefulness of smear preparation in liquid-based cytology methods. Asian Pac. J. Cancer Prev..

[130] Latiff L.A., Ibrahim Z., Pei C.P., Rahman S.A., Akhtari-Zavare M. (2015). Comparative Assessment of a Self-sampling Device and Gynecologist Sampling for Cytology and HPV DNA Detection in a Rural and Low Resource Setting: Malaysian Experience. Asian Pac. J. Cancer Prev..

[131] NCI SELF-CERV Pivotal Study: SELF-Collection for CERVical Cancer Screening. https://www.cancer.gov/about-cancer/treatment/clinical-trials/search/v?id=NCI-2024-01758.

[132] Nodjikouambaye Z.A., Compain F., Sadjoli D., Bouassa R.-S.M., Péré H., Veyer D., Robin L., Adawaye C., Tonen-Wolyec S., Moussa A.M. (2019). Accuracy of Curable Sexually Transmitted Infections and Genital Mycoplasmas Screening by Multiplex Real-Time PCR Using a Self-Collected Veil among Adult Women in Sub-Saharan Africa. Infect. Dis. Obstet. Gynecol..

[133] Aranda Flores C.E., Gomez Gutierrez G., Ortiz Leon J.M., Cruz Rodriguez D., Sørbye S.W. (2021). Self-collected versus clinician-collected cervical samples for the detection of HPV infections by 14-type DNA and 7-type mRNA tests. BMC Infect. Dis..

[134] Gradíssimo A., Burk R.D. (2017). Molecular tests potentially improving HPV screening and genotyping for cervical cancer prevention. Expert Rev. Mol. Diagn..

[135] Milanova V., Gomes M., Mihaylova K., Twelves J.L., Multmeier J., McMahon H., McCulloch H., Cuschieri K. (2025). Diagnostic accuracy of the Daye diagnostic tampon compared to clinician-collected and self-collected vaginal swabs for detecting HPV: A comparative study. J. Clin. Microbiol..

[136] Naseri S., Young S., Cruz G., Blumenthal P.D. (2022). Screening for High-Risk Human Papillomavirus Using Passive, Self-Collected Menstrual Blood. Obstet. Gynecol..

[137] Chakravarti P., Maheshwari A., Tahlan S., Kadam P., Bagal S., Gore S., Panse N., Deodhar K., Chaturvedi P., Dikshit R. (2022). Diagnostic accuracy of menstrual blood for human papillomavirus detection in cervical cancer screening: A systematic review. Ecancermedicalscience.

[138] Davies J.C., Sargent A., Pinggera E., Carter S., Gilham C., Sasieni P., Crosbie E.J. (2024). Urine high-risk human papillomavirus testing as an alternative to routine cervical screening: A comparative diagnostic accuracy study of two urine collection devices using a randomised study design trial. BJOG Int. J. Obstet. Gynaecol..

[139] Nilyanimit P., Chaithongwongwatthana S., Oranratanaphan S., Poudyal N., Excler J.-L., Lynch J., Vongpunsawad S., Poovorawan Y. (2024). Comparable detection of HPV using real-time PCR in paired cervical samples and concentrated first-stream urine collected with Colli-Pee device. Diagn. Microbiol. Infect. Dis..

[140] Gibert M.J., Sánchez-Contador C., Artigues G. (2023). Validity and acceptance of self vs conventional sampling for the analysis of human papillomavirus and Pap smear. Sci. Rep..

[141] Lorenzi N.P.C., Termini L., Filho A.L., Tacla M., de Aguiar L.M., Beldi M.C., Ferreira-Filho E.S., Baracat E.C., Soares-Júnior J.M. (2019). Age-related acceptability of vaginal self-sampling in cervical cancer screening at two university hospitals: A pilot cross-sectional study. BMC Public Health.

[142] Sabeena S., Kuriakose S., Binesh D., Abdulmajeed J., Dsouza G., Ramachandran A., Vijaykumar B., Aswathyraj S., Devadiga S., Ravishankar N. (2019). The Utility of Urine-Based Sampling for Cervical Cancer Screening in Low-Resource Settings. Asian Pac. J. Cancer Prev..

[143] Turner F., Drury J., Hapangama D.K., Tempest N. (2023). Menstrual Tampons Are Reliable and Acceptable Tools to Self-Collect Vaginal Microbiome Samples. Int. J. Mol. Sci..

[144] Teal Health FDA Puts Teal Health on an Accelerated Path to Market for Our At-Home Cervical Cancer Screening. https://www.getteal.com/post/fda-puts-teal-health-on-an-accelerated-path-to-market-for-our-at-home-cervical-cancer-screening.

[145] Shafaghmotlagh S. Cancer Research UK—Cancer News. 2024. Papcup: Could This at-Home HPV Test Make Cervical Screening Easier?. https://news.cancerresearchuk.org/2024/09/04/papcup-at-home-hpv-test-to-make-cervical-screening-smear-test-easier/.

[146] Asciutto K.C., Henningsson A.J., Borgfeldt H., Darlin L., Borgfeldt C. (2017). Vaginal and Urine Self-sampling Compared to Cervical Sampling for HPV-testing with the Cobas 4800 HPV Test. Anticancer Res..

[147] Ørnskov D., Jochumsen K., Steiner P.H., Grunnet I.M., Lykkebo A.W., Waldstrøm M. (2021). Clinical performance and acceptability of self-collected vaginal and urine samples compared with clinician-taken cervical samples for HPV testing among women referred for colposcopy. A cross-sectional study. BMJ Open.

[148] Liu X., Ning L., Fan W., Jia C., Ge L. (2024). Electronic Health Interventions and Cervical Cancer Screening: Systematic Review and Meta-Analysis. J. Med. Internet Res..

[149] Woo Y.L., Khoo S.P., Gravitt P., Hawkes D., Rajasuriar R., Saville M. (2022). The Implementation of a Primary HPV Self-Testing Cervical Screening Program in Malaysia through Program ROSE—Lessons Learnt and Moving Forward. Curr. Oncol..

[150] Olthof E.M.G., Aitken C.A., Siebers A.G., van Kemenade F.J., de Kok I.M.C.M. (2024). The impact of loss to follow-up in the Dutch organised HPV-based cervical cancer screening programme. Int. J. Cancer.

[151] Manley K., Patel A., Pawade J., Glew S., Hunt K., Villeneuve N., Mukonoweshuro P., Thompson S., Hoskins H., López-Bernal A. (2021). The use of biomarkers and HPV genotyping to improve diagnostic accuracy in women with a transformation zone type 3. Br. J. Cancer.

[152] Othman N.H., Zaki F.H.M., Hussain N.H.N., Yusoff W.Z.W., Ismail P. (2016). SelfSampling Versus Physicians’ Sampling for Cervical Cancer Screening Agreement of Cytological Diagnoses. Asian Pac. J. Cancer Prev..

[153] Verdoodt F., Dehlendorff C., Kjaer S.K. (2020). Dose-related Effectiveness of Quadrivalent Human Papillomavirus Vaccine Against Cervical Intraepithelial Neoplasia: A Danish Nationwide Cohort Study. Clin. Infect. Dis..

[154] Yeh P.T., E Kennedy C., de Vuyst H., Narasimhan M. (2019). Self-sampling for human papillomavirus (HPV) testing: A systematic review and meta-analysis. BMJ Glob. Health.

[155] Ouh Y.-T., Kim T.J., Ju W., Kim S.W., Jeon S., Kim S.-N., Kim K.G., Lee J.-K. (2024). Development and validation of artificial intelligence-based analysis software to support screening system of cervical intraepithelial neoplasia. Sci. Rep..

[156] Holmström O., Linder N., Kaingu H., Mbuuko N., Mbete J., Kinyua F., Törnquist S., Muinde M., Krogerus L., Lundin M. (2021). Point-of-Care Digital Cytology With Artificial Intelligence for Cervical Cancer Screening in a Resource-Limited Setting. JAMA Netw. Open.

[157] Kelly H., Benavente Y., Pavon M.A., De Sanjose S., Mayaud P., Lorincz A.T. (2019). Performance of DNA methylation assays for detection of high-grade cervical intraepithelial neoplasia (CIN2+): A systematic review and meta-analysis. Br. J. Cancer.

[158] Schreiberhuber L., Barrett J.E., Wang J., Redl E., Herzog C., Vavourakis C.D., Sundström K., Dillner J., Widschwendter M. (2024). Cervical cancer screening using DNA methylation triage in a real-world population. Nat. Med..

[159] Zhang L., Tan W., Yang H., Zhang S., Dai Y. (2022). Detection of Host Cell Gene/HPV DNA Methylation Markers: A Promising Triage Approach for Cervical Cancer. Front. Oncol..

[160] Chatzistamatiou K., Tsertanidou A., Moysiadis T., Mouchtaropoulou E., Pasentsis K., Skenderi A., Stamatopoulos K., Agorastos T. (2021). Comparison of different strategies for the triage to colposcopy of women tested high-risk HPV positive on self-collected cervicovaginal samples. Gynecol. Oncol..

[161] Song F., Du H., Wang C., Huang X., Wu R., CHIMUST Team (2020). The effectiveness of HPV16 and HPV18 genotyping and cytology with different thresholds for the triage of human papillomavirus-based screening on self-collected samples. PLoS ONE.

